# Differential Infectivities among Different Japanese Encephalitis Virus Genotypes in *Culex quinquefasciatus* Mosquitoes

**DOI:** 10.1371/journal.pntd.0005038

**Published:** 2016-10-05

**Authors:** Yan-Jang S. Huang, Susan M. Hettenbach, So Lee Park, Stephen Higgs, Alan D. T. Barrett, Wei-Wen Hsu, Julie N. Harbin, Lee W. Cohnstaedt, Dana L. Vanlandingham

**Affiliations:** 1 Department of Diagnostic Medicine/Pathobiology, College of Veterinary Medicine, Kansas State University, Manhattan, Kansas, United States of America; 2 Biosecurity Research Institute, Kansas State University, Manhattan, Kansas, United States of America; 3 Department of Pathology, University of Texas Medical Branch, Galveston, Texas, United States of America; 4 Sealy Center for Vaccine Development, University of Texas Medical Branch, Galveston, Texas, United States of America; 5 Department of Statistics, College of Arts and Sciences, Kansas State University, Manhattan, Kansas, United States of America; 6 Arthropod-borne Animal Disease Research Unit, Agriculture Research Service, United States Department of Agriculture, Manhattan, Kansas, United States of America; Aix Marseille University, Institute of Research for Development, and EHESP School of Public Health, FRANCE

## Abstract

During the last 20 years, the epidemiology of Japanese encephalitis virus (JEV) has changed significantly in its endemic regions due to the gradual displacement of the previously dominant genotype III (GIII) with clade b of GI (GI-b). Whilst there is only limited genetic difference distinguishing the two GI clades (GI-a and GI-b), GI-b has shown a significantly wider and more rapid dispersal pattern in several regions in Asia than the GI-a clade, which remains restricted in its geographic distribution since its emergence. Although previously published molecular epidemiological evidence has shown distinct phylodynamic patterns, characterization of the two GI clades has only been limited to *in vitro* studies. In this study, *Culex quinquefasciatus*, a known competent JEV mosquito vector species, was orally challenged with three JEV strains each representing GI-a, GI-b, and GIII, respectively. Infection and dissemination were determined based on the detection of infectious viruses in homogenized mosquitoes. Detection of JEV RNA in mosquito saliva at 14 days post infection indicated that *Cx*. *quinquefasciatus* can be a competent vector species for both GI and GIII strains. Significantly higher infection rates in mosquitoes exposed to the GI-b and GIII strains than the GI-a strain suggest infectivity in arthropod vectors may lead to the selective advantage of previously and currently dominant genotypes. It could thus play a role in enzootic transmission cycles for the maintenance of JEV if this virus were ever to be introduced into North America.

## Author Summary

Japanese encephalitis virus (JEV) is a zoonotic flavivirus, which is primarily transmitted by *Culex* species mosquitoes and a leading cause of pediatric encephalitis in Asia. JEV is also an important public health threat to countries outside the endemic region because collections of *Cx*. *quinquefasciatus* from around the world have demonstrated competence for the transmission of JEV and are capable of establishing enzootic transmission cycles between viremic avian and swine species. In the last two decades, the dominantly circulating genotype of JEV in endemic regions has experienced a significant shift (genotype III to Genotype I). It is unclear if the newly dominant circulating G1-b genotype can still be vectored by *Cx*. *quinquefasciatus*. In this study, *Cx*. *quinquefasciatus* collected from North America was demonstrated to be competent for the transmission of the newly dominant genotype. Different infectivities observed between the endemic strains and non-endemic strain provides the mechanistic knowledge of the selection and emergence of endemic genotypes after continuous viral evolution.

## Introduction

Japanese encephalitis virus (JEV) is a zoonotic flavivirus endemic in the Asian Pacific region. Transmission of JEV in endemic regions predominantly alternates between amplification hosts and *Culex tritaeniorhynchus* mosquitoes. Infected ardeid birds and pigs often develop high-titer viremia, which sustain subsequent transmission [1]. Similar to West Nile virus, another member in the JEV serocomplex, JEV has been known to utilize multiple species of mosquitoes for transmission and maintenance in nature [2]. In addition to the endemic vector *Cx*. *tritaeniorhynchus*, other species of mosquitoes have been demonstrated to be competent for transmission of JEV including: *Cx*. *fuscocephala*, *Cx*. *pipiens*, and *Cx*. *quinquefasciatus* [3–9]. In the process of its introduction to Australia, mainly in the northern islands, *Cx*. *annulirostris* was demonstrated to be a competent vector species for the first time [10]. Additional species belonging to the genus of *Aedes*, *Anopheles*, and *Armigeres* are also susceptible to JEV infection [2]. Although the majority of human infections are asymptomatic, childhood encephalitic diseases caused by JEV can often be lethal with a 20–30% mortality rate. Neurological disorders are reported in 30–50% of survivors [11]. Several vaccines have been developed and used for childhood immunization programs, which have substantially reduced the disease burden in the late 20^th^ century [12].

The evolution of JEV has led to at least five genotypes in epidemic and endemic regions in Asia. Strains in genotype III (GIII) have been reported to be the dominantly circulating genotype and associated with multiple outbreaks between 1935 and the 1990s in various countries; whereas, the first isolate of genotype I (GI) JEV strain did not occur until 1967 in Cambodia [13]. In the last two decades, the GI strains have been more frequently isolated and have resulted in the displacement of GIII strains in several countries in East Asia [14, 15] such that GI and GIII strains have been continuously circulating in Asia since the 1990s. Based on the isolates collected between the 1960s and 1990s, GI strains can be divided into two distinct clades, GI-a and GI-b [13, 16]. Both GI-a and GI-b originated from the endemic Southeast Asia region but showed distinct dispersal patterns and epidemiological characteristics. GI-a strains were first found in Thailand and Cambodia with its subsequent spread to Australia. In contrast to the restricted geographic distribution of GI-a strains, the GI-b strains have rapidly dispersed throughout the majority of East Asia and are ultimately responsible for the displacement of the GIII strains. Although previously published phylogenetic data established our fundamental knowledge of viral genetics and epidemiology, our understanding of the mechanisms responsible for the emergence of GI strains is very rudimentary. To date, only phenotypic studies have been undertaken using *in vitro* cell culture models [13]. A significant knowledge gap exists in the lack of experimental evidence derived from *in vivo* models to characterize the mechanism for genotype replacement.

Vector competence of various *Cx*. *quinquefasciatus* populations for JEV has been previously demonstrated by oral infection of GIII strains. Recently, our group has demonstrated that North American *Cx*. *quinquefasciatus* mosquitoes are competent vectors for the transmission of the JEV GIII Taira strain [17]. *Cx*. *quinquefasciatus* mosquitoes from Asia, Australia, and Brazil have also been shown to be competent vectors of JEV [3–5]. Collectively, these studies have demonstrated that JEV can be transmitted by *Cx*. *quinquefasciatus*. Despite the rapid emergence of GI-b strains, it has not been established whether or not *Cx*. *quinquefasciatus* can transmit GI strains. To determine vector competence of *Cx*. *quinquefasciatus* for GI strains and gain further insight into the emergence of GI strains, three JEV strains, each representing GI-a, GI-b and GIII, were orally administered to *Cx*. *quinquefasciatus*. Our results demonstrate distinct phenotypic differences between GI-a and GI-b strains in mosquitoes and suggest the difference in infectivity in competent mosquito species may be a critical determinant contributing to the emergence of GI-b strains.

## Materials and Methods

### Cells and viruses

African green monkey kidney Vero76 cells (source: Arthropod-Borne Animal Disease Research Unit, Agriculture Research Service, United States Department of Agriculture) and *Aedes albopictus* C6/36 cells (source: Arthropod-Borne Animal Disease Research Unit, Agriculture Research Service, United States Department of Agriculture) were maintained in L-15 media as previously described [18]. C6/36 cells were used to propagate virus stocks subsequently used in oral infection experiments. Vero76 cells were used for the detection of infectious viruses in the homogenized mosquito samples using the tissue culture 50% infectious dose (TCID_50_) method. Based on the published phylogenetic analysis, three strains of JEV were chosen as representatives for GI-a, GI-b and GIII [16, 19–21]. Strain KE-93-83 (source: existing virus culture collection in the laboratory of Dr. Alan D. T. Barrett, University of Texas Medical Branch) was used as a representative for GI-a. Prior to the study, it was passaged twice in Vero cells and once in C6/36 cells. Strain JE-91 (source: existing virus culture collection in the laboratory of Dr. Alan D. T. Barrett, University of Texas Medical Branch), originally isolated from mosquitoes collected in Korea in 1991 followed by one passage in Vero cells and one passage in C6/36 cells, was chosen as a representative for GI-b. The Taira strain (source: existing virus culture collection in the laboratory of Dr. Alan D. T. Barrett, University of Texas Medical Branch) originally derived from an infected human in an epidemic in Japan in 1948 represents GIII and was passaged twice in Vero cells. All three strains used in viremic blood meals were generated by a single passage in C6/36 cells at 28°C and harvested when greater than 80% of cytopathic effect was present.

### Mosquitoes and *per os* infection

*Culex quinquefasciatus* mosquitoes collected from Valdosta, GA (source: the laboratory of Dr. Mark Blackmore, Valdosta State University) were used in this study as previously described [17]. Mosquitoes were maintained in 12" cages with 10% sucrose ad libitum at 28°C. A 16hr:8hr light:dark photoregime was used for all experiments. For *per os* infection, eight-to-10-day-old female mosquitoes of generations below F_6_ were used. Prior to the infection, mosquitoes were deprived of sugar and water for 48 and 24 hours, respectively. Viremic blood meals were prepared by mixing virus stocks with defibrinated sheep blood (Colorado Serum, CO) and delivered through a Hemotek membrane feeding apparatus (Discovery Workshop) and cotton pledget for one hour. Mosquitoes were cold anesthetized on ice prior to sorting engorged mosquitoes. Three-to-5 engorged mosquitoes were immediately collected to assess the quantities of viruses ingested through the blood meals. Titers of viremic blood meals and three engorged mosquitoes are summarized in Table 1.

**Table 1:** Summary of the average titers of viremic blood meals and engorged mosquitoes; and infection, dissemination, and transmission rates.

JEV strains		KE-93-83	JE-91	Taira
Viremic blood meals (logTCID_50_/ml)		7.99±0.41	8.13±0.72	8.36±0.53
Engorged mosquitoes (logTCID_50_/ml)		4.36±0.89	4.81±1.23	4.63±0.57
Infection rate	7 d.p.i. *	43.9% (18/41)	57.6% (19/33)	95.1% (39/41)
	14 d.p.i. ^ǂ^	35.2% (19/54)	55.6% (25/45)	66.7% (44/66)
Dissemination rate	7 d.p.i.	23.1% (3/13)	30.0% (3/10)	8.3% (2/24)
	14 d.p.i.	16.7% (2/12)	28.6% (4/14)	32.1% (9/28)
Transmission rate	14 d.p.i.	5.3% (1/19)	8.0% (2/25)	6.8% (3/44)

Titers are shown as average titer ± standard deviation.

* indicates there was a significant difference between at least two of the three strains (*χ*^2^ = 25.49, *df* = 2, *p* < 0.05). Tukey-type multiple comparison showed the significantly higher infection rate was observed in mosquitoes infected by Taira strain than those infected by KE-93-83 strain and JE-91 strain.

^ǂ^ indicates there was a significant difference between at least two of the three strains (*χ*^2^ = 11.95, *df* = 2, *p* < 0.05). Tukey-type multiple comparison showed the significantly higher infection rate was observed in mosquitoes infected by Taira strain than those infected by KE-93-83 strain.

Mosquitoes were collected at 7 and 14 days post-infection (d.p.i.) by mechanical aspiration. Dissections were performed to separate the body (abdomen) section and secondary tissues (head, wings, and legs) of individual mosquitoes. Whole mosquitoes were also collected to assess the growth kinetics. All tissue samples were collected in 1ml L-15 medium supplemented with amphotericin B and sodium deoxycholate at 1 and 0.8 μg/ml, respectively. Homogenization and titration of samples were performed using previously published methods [18].

At 14 d.p.i., saliva of each mosquito was collected prior to dissection [18]. Viral RNA was extracted with the QIAamp viral RNA extraction kit (Qiagen) and reverse transcribed by Superscript III reverse transcriptase (Life Technologies) with the reverse primer (Integrated DNA Technologies) prMR3 (5'-CATGAGGTATCGCGTGGC-3'). cDNA was amplified by Taq DNA polymerase (New England BioLabs) using the semi-nested PCR cycles described by Johansen *et al*. [22]. Primer sets target the conserved region between the capsid and pre-membrane genes. The outer set of primers were forward primer FV128 (5'-CCGGGCTGTCAATATGCT-3') and reverse primer prMR3. The inner set of primers were forward primer FV128 and reverse primer JE659 (5'-CACCAGCAATCCACGTCCTC-3').

### Statistical analysis

Infection status of orally challenged mosquitoes was determined by the detection of infectious viruses from homogenized samples. Infection rate was calculated by dividing the number of infected mosquitoes over the number of mosquitoes tested. Disseminated infection was defined by the detection of infectious viruses from homogenized secondary tissues among dissected mosquitoes. Dissemination rates were determined by the numbers of dissected mosquitoes containing positive secondary tissues divided by the number of infected mosquitoes dissected. Viral transmission was demonstrated by the presence of viral RNA in the saliva detected by semi-nested RT-PCR. Transmission rate was calculated by dividing the numbers of mosquitoes containing positive saliva by the numbers of infected mosquitoes. Infection, dissemination and transmission rates were compared among three JEV strains tested by Chi-square test or Fisher’s exact test coupled with a Tukey-type multiple comparison test. Analysis of growth kinetics was performed by comparing the titers of whole mosquitoes collected at 7 and 14 d.p.i. with Friedman’s two-way nonparametric analysis of variance. All statistical analyses were conducted using the SAS (Statistical Analysis System) software (version 9.4, Cary, NC).

## Results

### Infection of JEV Japanese encephalitis virus in *Culex quinquefasciatus*

Infection data following oral challenge with different JEV strains are summarized in Table 1. At 7 d.p.i., there was a significant difference in the infection rates among the three strains tested (Chi-square = 25.49, df = 2, *p*<0.001) with the highest infection rate observed in mosquitoes exposed to the GIII Taira strain (95.1%, 39/41) and the lowest infection rate observed among mosquitoes exposed to the GI-a KE-93-83 strain (43.9%, 18/41). The infection rate of the GIII Taira strain group was also significantly higher than the infection rate of the GI-b JE-91 strain (57.6%, 19/33). No statistical difference was found in the infection rates between the KE-93-83 strain and the JE-91 strain.

Infection rates for the three JEV strains were significantly different at 14 d.p.i. (Chi-square = 11.95, df = 2, *p* = 0.003). The two representatives of the endemic genotypes GI-b and GIII showed no statistical difference; the JE-91 strain (55.6%, 25/45) and the Taira strain (66.7%, 44/66). Interestingly, the representative of the non-endemic GI-a, the KE-93-83 strain, maintained the lowest infection rate among the three strains. Tukey type multiple comparison showed a significantly lower infection rate (35.2%, 19/54) than the Taira strain (*p*<0.05). Overall, the KE-93-83 strain, the representative strain of non-endemic GI-a, showed the lower infectivity than the other two strains, which are the strains representing the endemic GI-b and GIII.

### Dissemination of Japanese encephalitis virus in infected mosquitoes

Based on the observation that JEV strains can successfully establish infections, we investigated whether or not the infection will ultimately lead to viral dissemination. Titration of secondary tissues demonstrated that infection of all three strains led to dissemination (Table 1). There were no statistical differences in the dissemination rates among the three strains tested at 7 d.p.i. (Fisher’s Exact test, *p* = 0.231) or 14 d.p.i (Fisher’s Exact test, *p* = 0.664). At 7 d.p.i., the dissemination rate of the KE-93-83 strain was 23.1% (3/13). Comparable dissemination rates were observed among the mosquitoes infected by the JE-91 strain (30.0%, 3/10) and the Taira strain (8.3%, 2/24). Dissemination rates were also similar at 14 d.p.i. among the KE-93-83 strain (16.7%, 2/12), the JE-91 strain (28.6%, 4/14), and the Taira strain (32.1%, 9/28). The results suggest that all three JEV strains in this study were able to disseminate into secondary tissues after the establishment of infection.

### Detection of viral RNA in mosquito saliva

Detection of JEV viral RNA in the saliva of virus-infected mosquitoes was achieved by semi-nested RT-PCR indicating the capacity of viral transmission among *Cx*. *quinquefasciatus* tested in this study as shown in Table 1. Among the mosquitoes infected by the KE-93-83 strain, 5.3% (1/19) of saliva samples tested were positive for the presence of JEV viral RNA. Similar results were also observed in mosquitoes infected by the JE-91 strain (8.0%, 2/25) and the Taira strain (6.8%, 3/44). There was no demonstrable difference in the transmission rates among the three strains tested in this study (Fisher’s Exact test, *p* = 0.999) indicating North American *Cx*. *quinquefasciatus* can serve as competent vectors for GI-a, GI-b and GIII JEV strains.

### Replication kinetics of JEV in infected mosquitoes

**Fig 1:**
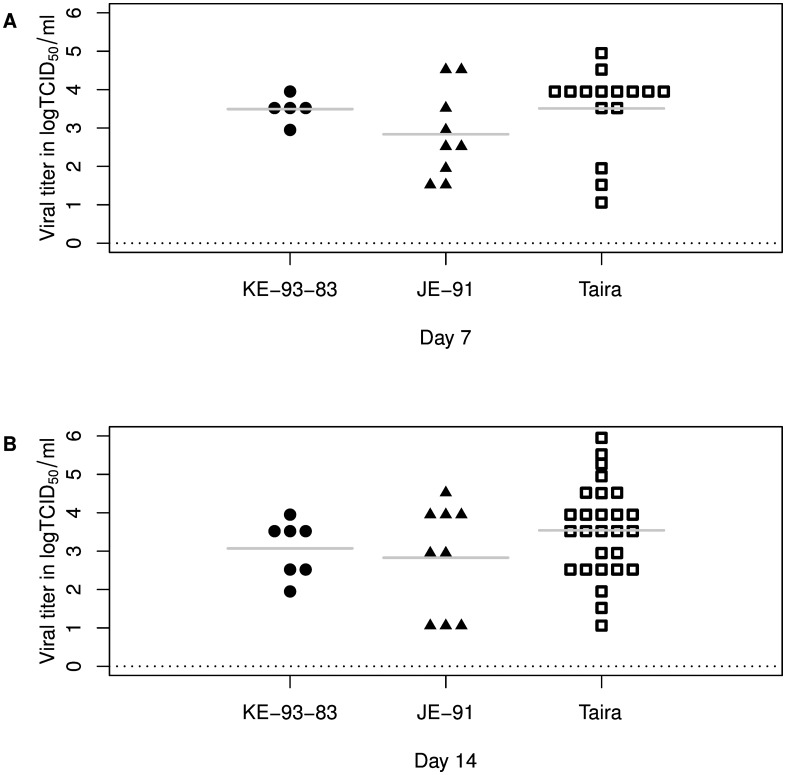
Viral titers of JEV-infected mosquitoes at 7 (A) and 14 (B) days post infection. The horizontal bar represents the average titers of whole mosquitoes infected by each strain of JEV.

As dissemination and transmission require viral replication in various tissues in infected mosquitoes, the replication kinetics of three JEV strains was analyzed by the titration of infected whole mosquitoes. The results are summarized in Fig 1. At 7 d.p.i., the average virus infectivity titer in mosquitoes infected by the KE-93-83 strain was 3.5 logTCID_50_/ml (*n* = 5) whereas for the JE-91 and Taira strains it was 2.8 (*n* = 9) and 3.5 (*n* = 15) logTCID_50_/ml, respectively. The average infectivity titer in mosquitoes infected by the KE-93-83 strain at 14 d.p.i. was 3.1 logTCID_50_/ml (*n* = 7) while the JE-91 and Taira strains maintained average titers of 2.8 (n = 9) and 3.5 (*n* = 37) logTCID_50_/ml, respectively. Friedman’s two-way nonparametric analysis of variance showed there was no significant difference in the average titers of infected mosquitoes among the three different strains (*p* = 0.174) at 7 or 14 d.p.i.

## Discussion

Our observations improve our understanding of the potential for the establishment of enzootic transmission cycles by the GI-b strains of JEV in North America. Although the GI-b strains have gradually become the dominant genotype in Asia, since 1990s, there is no information available to assess the risk of it establishing enzootic transmission cycles in North America or other regions, where JEV has not been reported. Our results support the potential role of North American *Cx*. *quinquefasciatus* as a competent vector for the newly emerging GI-b strains. With the additional evidence showing that North American avian species can develop viremia after being challenged with a GI strain [23], it is reasonable to conclude that JEV remains as an important human and veterinary public health threat after the shift of the dominant genotype from GIII to GI-b.

To the best of our knowledge, this is also the first report identifying phenotypic difference among different JEV genotypes *in vivo*. Significantly higher infection rates were observed at 7 and 14 d.p.i. in *Cx*. *quinquefasciatus* challenged by endemic GI-b and GIII strains of JEV; whereas, the non-endemic GI-a strain consistently showed significantly lower infection rates. Because *Cx*. *quinquefasciatus* does not belong to the *Cx*. *vishnui* complex, which is more directly related to the endemic transmission of JEV in nature, the results should be interpreted with caution. However, the importance in characterizing JEV in *Cx*. *quinquefasciatus* should not be overlooked because of its role as a source of JEV isolates in nature and the published evidence demonstrating its vector competence for JEV in multiple laboratory studies [3, 8, 17, 24–26]. Given the anticipated high mortality from transporting eggs of *Cx*. *tritaeniorhynchus* from Asia [13], using *Cx*. *quinquefasciatus* is an acceptable substitute for characterization of JEV *in vivo*. The results suggest that endemic GI-b and GIII strains of JEV are more infectious to *Cx*. *quinquefasciatus* through oral infection than GI-a strains, which have not become endemic since its emergence. This finding agrees with the epidemiological observations and directly contributes to much needed knowledge as to why GI-b strains of JEV can emerge after continuous evolution.

Previously, superior multiplication kinetics were reported for the GI-b JE-91 strain in C6/36 *Ae*. *albopictus* cells compared to GIII Tiara with GI-a KE-93-83 having the lowest multiplication kinetics of the three virus strains. It was suggested this might result in a selective advantage explaining the emergence of GI-b strains and the subsequent displacement of GIII strains [13]. An alternative hypothesis that could explain the emergence of the GI-b strains is the subtle difference in the amino acid sequences in the NS5 RNA-dependent RNA polymerase between the GI and GIII strains may result in an increase in replication efficiency [27]. These two hypotheses are not mutually exclusive, but previous studies showed the three virus strains had indistinguishable multiplication kinetics in Duck embryo fibroblast cells [13]. A comparative analysis of the multiplication kinetics of the GI-b and GIII strains was previously performed in North American avian species and demonstrated that multiplication kinetics of the GI-b strain was at least as high as the GIII strain and in most cases multiplied to higher titer [23]. Detection of viremia in North American avian species suggests the possibility of establishment of enzootic transmission by providing sources of infectious blood meals for competent mosquitoes. Higher viremic titer in infected avian species caused by the GI-b strain further suggests the newly emerging GI-b strains can have higher epidemic potential than the previously dominant GIII strains. Our 14 d.p.i oral infection data for *Cx*. *quinquefasciatus* in this study showed the GI-b strain was as infectious and disseminated similarly to the GIII strain whereas the GI-a strain did not. Multiplication kinetics between the GI-b strain and GIII strain also did not differ significantly and subsequently resulted in different titers of infected mosquitoes. The lack of a distinguishable difference between the GI-b and GIII strains in our study may be due to the difference between the *in vitro* and *in vivo* experimental conditions utilizing C6/36 cells and orally infected *Cx*. *quinquefasciatus*, respectively. Therefore, with respect to mosquito data, we did not observe a selective advantage of the GI-b strains over the GIII strains and so cannot conclude the displacement of the GIII strains by the GI-b strains in various endemic countries is due to differences in infectivity for vectors, rather enhanced infection of mosquitoes by GI-b compared to GI-a viruses together with higher viremias than GIII viruses in birds may have led to the selective advantage of the GI-b viruses.

Through *per os* infection, our and others’ studies demonstrated *Cx*. *quinquefasciatus*, which is commonly present in the sub-tropical region of the American continent, can be competent enzootic vectors for members of the JEV serocomplex such as Saint Louis encephalitis virus (SLEV) and West Nile virus (WNV) [28, 29]. In contrast to our observation on the infection of endemic JEV strains in *Cx*. *quinquefasciatus*, oral ingestion of WNV resulted in comparable or significantly higher infection and dissemination rates regardless of genotypes used in the studies [28, 30–35]. Similar to our observations on endemic strains of JEV in this study, previously published studies also demonstrated strains of SLEV with higher epidemic potential are more infectious than non-epidemic strains when orally delivered to *Cx*. *quinquefasciatus* collected from Argentina [29]. A more recently published study also demonstrated the difference in infectivity between genotype III and V of SLEV in *Cx*. *quinquefasciatus* [36]. Based on the infection study performed with *Cx*. *quinquefasciatus* collected from Gainesville, FL, the higher infection rate of SLEV (93–100%) was achieved with relatively low viremic titers compared to JEV challenged at higher viral titers in this study. However, there was no distinguishable difference between SLEV and endemic strains of JEV in the capacity for dissemination into secondary tissues from the infected midguts [28].

In addition to the evidence supporting the potential role of *Cx*. *quinquefasciatus* as a competent vector, it is important to keep in mind that there are multiple species of mosquitoes found in North America, which have been previously demonstrated to be competent for the transmission of JEV and other related flaviviruses [37]. As observed with the process of WNV becoming endemic in North American since 1999, it is certainly favorable for arboviruses to utilize multiple species of mosquitoes as enzootic vectors in order to establish its transmission cycles and achieve viral maintenance in adverse climatic conditions, especially winter [38, 39]. Further evaluation on other medically important *Culex* species mosquitoes will be critical for understanding the relative risk of the introduction of JEV and the establishment of its enzootic transmission cycles.

## References

[1] van den Hurk AF, Ritchie SA, Mackenzie JS. Ecology and geographical expansion of Japanese encephalitis virus. Annual review of entomology. 2009;54:17–35. 10.1146/annurev.ento.54.110807.090510 . 19067628

[2] Rosen L. The natural history of Japanese encephalitis virus. Annual review of microbiology. 1986;40:395–414. 10.1146/annurev.mi.40.100186.002143 . 2877613

[3] van den Hurk AF, Nisbet DJ, Hall RA, Kay BH, MacKenzie JS, Ritchie SA. Vector competence of Australian mosquitoes (Diptera: Culicidae) for Japanese encephalitis virus. Journal of medical entomology. 2003;40(1):82–90. 10.1603/0022-2585-40.1.82 . 12597658

[4] Mackenzie-Impoinvil L, Impoinvil DE, Galbraith SE, Dillon RJ, Ranson H, Johnson N, et al Evaluation of a temperate climate mosquito, Ochlerotatus detritus (= Aedes detritus), as a potential vector of Japanese encephalitis virus. Medical and veterinary entomology. 2015;29(1):1–9. 10.1111/mve.12083 . 25087926

[5] Turell MJ, Mores CN, Dohm DJ, Lee WJ, Kim HC, Klein TA. Laboratory transmission of Japanese encephalitis, West Nile, and Getah viruses by mosquitoes (Diptera: Culicidae) collected near Camp Greaves, Gyeonggi Province, Republic of Korea 2003. Journal of medical entomology. 2006;43(5):1076–81. 10.1093/jmedent/43.5.1076 . 17017248

[6] Doi R. Studies on the mode of development of Japanese encephalitis virus in some groups of mosquitoes by the fluorescent antibody technique. The Japanese journal of experimental medicine. 1970;40(2):101–15. . 4318556

[7] Muangman D, Edelman R, Sullivan MJ, Gould DJ. Experimental transmission of Japanese encephalitis virus by Culex fuscocephala. Am J Trop Med Hyg. 1972;21(4):482–6. . 4340450 10.4269/ajtmh.1972.21.482

[8] Weng MH, Lien JC, Wang YM, Lin CC, Lin HC, Chin C. Isolation of Japanese encephalitis virus from mosquitoes collected in Northern Taiwan between 1995 and 1996. Journal of microbiology, immunology, and infection = Wei mian yu gan ran za zhi. 1999;32(1):9–13. . 11561572

[9] Mourya DT, Mishra AC. Antigen distribution pattern of Japanese encephalitis virus in Culex tritaeniorhynchus, C. vishnui & C. pseudovishnui. The Indian journal of medical research. 2000;111:157–61. . 10943067

[10] Ritchie SA, Phillips D, Broom A, Mackenzie J, Poidinger M, van den Hurk A. Isolation of Japanese encephalitis virus from Culex annulirostris in Australia. Am J Trop Med Hyg. 1997;56(1):80–4. . 9063367 10.4269/ajtmh.1997.56.80

[11] Campbell GL, Hills SL, Fischer M, Jacobson JA, Hoke CH, Hombach JM, et al Estimated global incidence of Japanese encephalitis: a systematic review. Bulletin of the World Health Organization. 2011;89(10):766–74, 74A–74E. 10.2471/BLT.10.085233 22084515 PMC3209971

[12] Tsai TF. New initiatives for the control of Japanese encephalitis by vaccination: minutes of a WHO/CVI meeting, Bangkok, Thailand, 13–15 October 1998. Vaccine. 2000;18 Suppl 2:1–25. 10.1016/S0264-410X(00)00037-2 . 10821969

[13] Schuh AJ, Ward MJ, Leigh Brown AJ, Barrett AD. Dynamics of the emergence and establishment of a newly dominant genotype of Japanese encephalitis virus throughout Asia. Journal of virology. 2014;88(8):4522–32. 10.1128/JVI.02686-13 24501419 PMC3993778

[14] Do LP, Bui TM, Hasebe F, Morita K, Phan NT. Molecular epidemiology of Japanese encephalitis in northern Vietnam, 1964–2011: genotype replacement. Virology journal. 2015;12:51 10.1186/s12985-015-0278-4 25889499 PMC4417254

[15] Su CL, Yang CF, Teng HJ, Lu LC, Lin C, Tsai KH, et al Molecular epidemiology of Japanese encephalitis virus in mosquitoes in Taiwan during 2005–2012. PLoS neglected tropical diseases. 2014;8(10):e3122 10.1371/journal.pntd.0003122 25275652 PMC4183467

[16] Schuh AJ, Ward MJ, Brown AJ, Barrett AD. Phylogeography of Japanese encephalitis virus: genotype is associated with climate. PLoS neglected tropical diseases. 2013;7(8):e2411 10.1371/journal.pntd.0002411 24009790 PMC3757071

[17] Huang YJ, Harbin JN, Hettenbach SM, Maki E, Cohnstaedt LW, Barrett AD, et al Susceptibility of a North American Culex quinquefasciatus to Japanese Encephalitis Virus. Vector borne and zoonotic diseases. 2015;15(11):709–11. 10.1089/vbz.2015.1821 . 26565775

[18] Vanlandingham DL, Schneider BS, Klingler K, Fair J, Beasley D, Huang J, et al Real-time reverse transcriptase-polymerase chain reaction quantification of West Nile virus transmitted by Culex pipiens quinquefasciatus. The American journal of tropical medicine and hygiene. 2004;71(1):120–3. Epub 2004/07/09. . 15238700

[19] Schuh AJ, Guzman H, Tesh RB, Barrett AD. Genetic diversity of Japanese encephalitis virus isolates obtained from the Indonesian archipelago between 1974 and 1987. Vector borne and zoonotic diseases. 2013;13(7):479–88. 10.1089/vbz.2011.0870 23590316 PMC3700436

[20] Schuh AJ, Li L, Tesh RB, Innis BL, Barrett AD. Genetic characterization of early isolates of Japanese encephalitis virus: genotype II has been circulating since at least 1951. The Journal of general virology. 2010;91(Pt 1):95–102. 10.1099/vir.0.013631-0 19776238 PMC2885061

[21] Schuh AJ, Tesh RB, Barrett AD. Genetic characterization of Japanese encephalitis virus genotype II strains isolated from 1951 to 1978. The Journal of general virology. 2011;92(Pt 3):516–27. 10.1099/vir.0.027110-0 21123550 PMC3081233

[22] Johansen CA, Hall RA, van den Hurk AF, Ritchie SA, Mackenzie JS. Detection and stability of Japanese encephalitis virus RNA and virus viability in dead infected mosquitoes under different storage conditions. Am J Trop Med Hyg. 2002;67(6):656–61. . 12518858 10.4269/ajtmh.2002.67.656

[23] Nemeth N, Bosco-Lauth A, Oesterle P, Kohler D, Bowen R. North American birds as potential amplifying hosts of Japanese encephalitis virus. Am J Trop Med Hyg. 2012;87(4):760–7. 10.4269/ajtmh.2012.12-0141 22927494 PMC3516332

[24] Nitatpattana N, Apiwathnasorn C, Barbazan P, Leemingsawat S, Yoksan S, Gonzalez JP. First isolation of Japanese encephalitis from Culex quinquefasciatus in Thailand. The Southeast Asian journal of tropical medicine and public health. 2005;36(4):875–8. . 16295539

[25] Vythilingam I, Oda K, Mahadevan S, Abdullah G, Thim CS, Hong CC, et al Abundance, parity, and Japanese encephalitis virus infection of mosquitoes (Diptera:Culicidae) in Sepang District, Malaysia. Journal of medical entomology. 1997;34(3):257–62. . 9151487 10.1093/jmedent/34.3.257

[26] Mourya DT, Ilkal MA, Mishra AC, Jacob PG, Pant U, Ramanujam S, et al Isolation of Japanese encephalitis virus from mosquitoes collected in Karnataka state, India from 1985 to 1987. Transactions of the Royal Society of Tropical Medicine and Hygiene. 1989;83(4):550–2. 10.1016/0035-9203(89)90288-5 . 2575809

[27] Han N, Adams J, Chen P, Guo ZY, Zhong XF, Fang W, et al Comparison of genotypes I and III in Japanese encephalitis virus reveals distinct differences in their genetic and host diversity. Journal of virology. 2014;88(19):11469–79. 10.1128/JVI.02050-14 25056890 PMC4178791

[28] Pesko K, Mores CN. Effect of sequential exposure on infection and dissemination rates for West Nile and St. Louis encephalitis viruses in Culex quinquefasciatus. Vector borne and zoonotic diseases. 2009;9(3):281–6. 10.1089/vbz.2007.0281 19492941 PMC2719847

[29] Mitchell CJ, Gubler DJ, Monath TP. Variation in infectivity of Saint Louis encephalitis viral strains for Culex pipiens quinquefasciatus (Diptera: Culicidae). Journal of medical entomology. 1983;20(5):526–33. 10.1093/jmedent/20.5.526 . 6315940

[30] Richards SL, Anderson SL, Lord CC. Vector competence of Culex pipiens quinquefasciatus (Diptera: Culicidae) for West Nile virus isolates from Florida. Tropical medicine & international health: TM & IH. 2014;19(5):610–7. 10.1111/tmi.12277 24898274 PMC4101994

[31] Anderson SL, Richards SL, Tabachnick WJ, Smartt CT. Effects of West Nile virus dose and extrinsic incubation temperature on temporal progression of vector competence in Culex pipiens quinquefasciatus. Journal of the American Mosquito Control Association. 2010;26(1):103–7. 10.2987/09-5926.1 20402358 PMC2858365

[32] McGee CE, Shustov AV, Tsetsarkin K, Frolov IV, Mason PW, Vanlandingham DL, et al Infection, dissemination, and transmission of a West Nile virus green fluorescent protein infectious clone by Culex pipiens quinquefasciatus mosquitoes. Vector borne and zoonotic diseases. 2010;10(3):267–74. 10.1089/vbz.2009.0067 19619041 PMC2883532

[33] Reisen WK, Barker CM, Fang Y, Martinez VM. Does variation in Culex (Diptera: Culicidae) vector competence enable outbreaks of West Nile virus in California? Journal of medical entomology. 2008;45(6):1126–38. 10.1093/jmedent/45.6.1126 . 19058638

[34] Vanlandingham DL, McGee CE, Klinger KA, Vessey N, Fredregillo C, Higgs S. Relative susceptibilties of South Texas mosquitoes to infection with West Nile virus. The American journal of tropical medicine and hygiene. 2007;77(5):925–8. Epub 2007/11/07. . 17984355

[35] Vanlandingham DL, McGee CE, Klingler KA, Galbraith SE, Barrett AD, Higgs S. Short report: comparison of oral infectious dose of West Nile virus isolates representing three distinct genotypes in Culex quinquefasciatus. The American journal of tropical medicine and hygiene. 2008;79(6):951–4. Epub 2008/12/05. ; PubMed Central PMCID: PMCPmc2699256. 19052310 PMC2699256

[36] Diaz LA, Flores FS, Beranek M, Rivarola ME, Almiron WR, Contigiani MS. Transmission of endemic St Louis encephalitis virus strains by local Culex quinquefasciatus populations in Cordoba, Argentina. Transactions of the Royal Society of Tropical Medicine and Hygiene. 2013;107(5):332–4. 10.1093/trstmh/trt023 . 23474474

[37] Reeves WC, Hammon WM, Technical Assistance of Griselda GW, Carlos E. Laboratory Transmission of Japanese B Encephalitis Virus by Seven Species (Three Genera) of North American Mosquitoes. The Journal of experimental medicine. 1946;83(3):185–94. 10.1084/jem.83.3.185 19871524 PMC2135585

[38] Huang YJ, Higgs S, Horne KM, Vanlandingham DL. Flavivirus-Mosquito Interactions. Viruses. 2014;6(11):4703–30. 10.3390/v6114703 25421894 PMC4246245

[39] Granwehr BP, Lillibridge KM, Higgs S, Mason PW, Aronson JF, Campbell GA, et al West Nile virus: where are we now? The Lancet infectious diseases. 2004;4(9):547–56. 10.1016/S1473-3099(04)01128-4 . 15336221

